# Contraindications to Lateral Extra-Articular Tenodesis: A Systematic Review

**DOI:** 10.3390/jcm15082821

**Published:** 2026-04-08

**Authors:** Jakub Erdmann, Jan Czerwiński, Adam Kwapisz, Maria Zabrzyńska, Gazi Huri, Piotr Walus, Jan Zabrzyński

**Affiliations:** 1 Department of Orthopaedics and Traumatology, Faculty of Medicine, Collegium Medicum in Bydgoszcz, Nicolaus Copernicus University in Toruń, 85-092 Bydgoszcz, Poland; erdmann.jakub@gmail.com (J.E.); zabrzynski@gmail.com (J.Z.); 2 Clinic of Orthopedics and Pediatric Orthopedics, Medical University of Łódź, 92-213 Łódź, Poland; adam.kwapisz@gmail.com; 3 Department of Family Medicine, Collegium Medicum, Nicolaus Copernicus University in Toruń, 85-094 Bydgoszcz, Poland; maria.zabrzynska@gmail.com; 4 Department of Orthopaedic and Sports Medicine, Hospital Doha, Doha 29222, Qatar; gazihuri@yahoo.com; 5 Department of Orthopaedics and Traumatology, Faculty of Medicine, Jan Kochanowski University in Kielce, 25-001 Kielce, Poland; walus.md@gmail.com

**Keywords:** lateral extra-articular tenodesis, LET, contraindications, ACL reconstruction, anterolateral ligament

## Abstract

**Background**: Lateral extra-articular tenodesis (LET) is a surgical procedure that is additionally implemented in concurrent anterior cruciate ligament reconstruction (ACLR). Although numerous articles have addressed the use of LET in conjunction with ACLR, few definitive contraindications were identified. Given the scarcity of literature evaluating contraindications for LET modality, this study aimed to systematically review the reported contraindications of this procedure in the context of concurrent ACLR. **Methods**: The searched key terms: (extra-articular OR extraarticular) AND (tenodesis OR plasty OR augmentation OR procedure or reconstruction OR reconstructive OR surgical OR surgery OR technique) AND (ACL OR anterior cruciate ligament), with no publication date restrictions in PubMed, ScienceDirect, Cochrane Central, Web of Science, and Embase databases. We included clinical human studies, with levels of evidence I–III and in the English language. **Results**: The analysis evaluated fourteen articles published between 2012 and 2024. Level III evidence was found in the majority of studies (*n* = 9) and Level I evidence was found in the rest (*n* = 5). The majority of the included articles were retrospective (*n* = 8) and there were also prospective studies (*n* = 6). The articles reviewed showed that articular cartilage damage and concomitant injuries to other knee ligaments, alongside ACL injury, are the most frequently mentioned. **Conclusions**: This is the first study that systematized the contraindications for the LET procedure in ACLR. The contraindications remain unclear; however, the following may be highlighted: articular cartilage damage and injury to another ligament in the knee, in addition to ACL injury.

## 1. Introduction

Anterior cruciate ligament reconstruction (ACLR) is one of the most commonly performed surgical techniques in sports medicine [1]. While it provides good clinical results, residual rotation laxity may be present [2,3]. However, one of the methods to limit excessive rotational instability is lateral extra-articular tenodesis (LET) [4]. This technique typically involves using a strip of the iliotibial band (ITB), passing it either over or under the fibular collateral ligament (FCL), and attaching it to the lateral femoral condyle. This non-anatomical fixation, when combined with ACLR, has proven to be a highly effective strategy for preventing anterolateral rotatory laxity of the knee, as demonstrated in cadaveric studies [4,5].

The technique was first described by Lemaire in 1967 and was initially used separately to treat ACL-deficient knees in the absence of intra-articular reconstruction techniques [6]. Nevertheless, thanks to the publication by Claes et al., the structure of the anterolateral ligament (ALL) has been detailed, along with its function in cadaveric studies [7]. The findings suggest that it functions as a secondary stabilizer to the ACL by resisting anterior translation and internal rotation of the tibia [8]. Consequently, there has been renewed interest in the LET technique, a new trend in knee surgery, supported by modern scientific findings [9]. In addition, the use of LET, which has a graft orientation that is more optimal for resisting tibial internal rotation, also resists the anterior translation better compared to ALL [10,11].

The topic of LET is gaining significant attention and continues to evolve. Despite the abundance of the literature on the subject, some specific aspects, such as the best technique and contraindications, remain unclear [12]. New studies continue to highlight the advantages of the LET technique, including improved rotational stabilization and reduced load on the ACL [13,14]. However, not all patients with ACL injuries are suitable candidates for lateral tenodesis [12]. There remain concerns as to whether LET may increase the risk of osteoarthritis in the lateral compartment of the knee joint [15,16]. Furthermore, emerging concepts in the treatment of knee ligament injuries, such as the epiligament, may influence future therapeutic strategies for managing these types of knee joint injuries [17]. To facilitate better patient selection and clinical decision-making, this systematic review seeks to identify and categorize the contraindications for LET as reported in the current literature.

## 2. Materials and Methods

### 2.1. Search Strategy

A comprehensive literature search was executed across several major databases, including PubMed, Cochrane Central, ScienceDirect, Web of Science, and Embase, to identify publications relevant to contraindications for LET. This investigation, performed independently by three authors (initials blinded for review) in October 2024, utilized a combination of keywords, including the following: (extra-articular OR extraarticular) AND (tenodesis OR plasty OR augmentation OR procedure OR reconstruction OR reconstructive OR surgical OR surgery OR technique) AND (ACL OR anterior cruciate ligament). No temporal filters were applied to the search. Furthermore, the reference lists of retrieved articles were manually screened for additional relevant studies. The review process adhered to the Preferred Reporting Items for Systematic Reviews and Meta-Analyses (PRISMA) standards [18] (Supplementary Materials). The protocol was registered with PROSPERO (CRD42023428461).

### 2.2. Eligibility Assessment

Following the initial database search, three independent reviewers (initials blinded for review) conducted a multi-stage screening of titles, abstracts, and full-text articles to evaluate their relevance to LET contraindications. Eligibility was restricted to clinical human studies (Levels I–III) published in English. We excluded case reports, reviews, correspondence, conference abstracts, and studies with insufficient or extraneous data (Levels IV and V). Additional exclusion criteria involved basic science research, investigations focusing on joints other than the knee, anatomical or radiographic studies, and animal models. Articles failing to provide a clear description of surgical indications or contraindications were also omitted. Discrepancies during the selection process were resolved through consultation with two senior authors specializing in evidence-based medicine (initials blinded for review).

### 2.3. Data Extraction

Data from the finalized list of articles were gathered by three independent authors (initials blinded for review). The extracted information encompassed several key parameters: year of publication, geographical origin, level of evidence, and study design. Additionally, patient-specific data, such as cohort size, mean age, and sex distribution, were recorded alongside the primary outcomes regarding LET contraindications. The level of evidence of the included studies was assessed according to the Oxford Centre for Evidence-Based Medicine (OCEBM) classification

### 2.4. Risk-of-Bias Assessment

To evaluate the methodological quality of the included studies, we utilized the Risk of Bias tool developed by the Cochrane Collaboration [19]. Each study was appraised across several critical domains, including selection, performance, detection, attrition, and reporting bias. These categories were classified as having a low, high, or uncertain risk based on the established criteria. This assessment was conducted independently by three reviewers, with any discrepancies being resolved through consensus.

### 2.5. Data Synthesis

Given the heterogeneity of the included studies, a formal meta-analysis was not performed. Instead, we used a descriptive ‘vote-counting’ approach to synthesize the data, tracking the frequency with which each contraindication was reported across the literature.

To better understand these patterns, we also compared the findings based on the quality of the evidence. We looked at whether high-level evidence (Level I trials) showed more or less consensus on specific contraindications compared to lower-level studies (Level III).

## 3. Results

**Figure 1:**
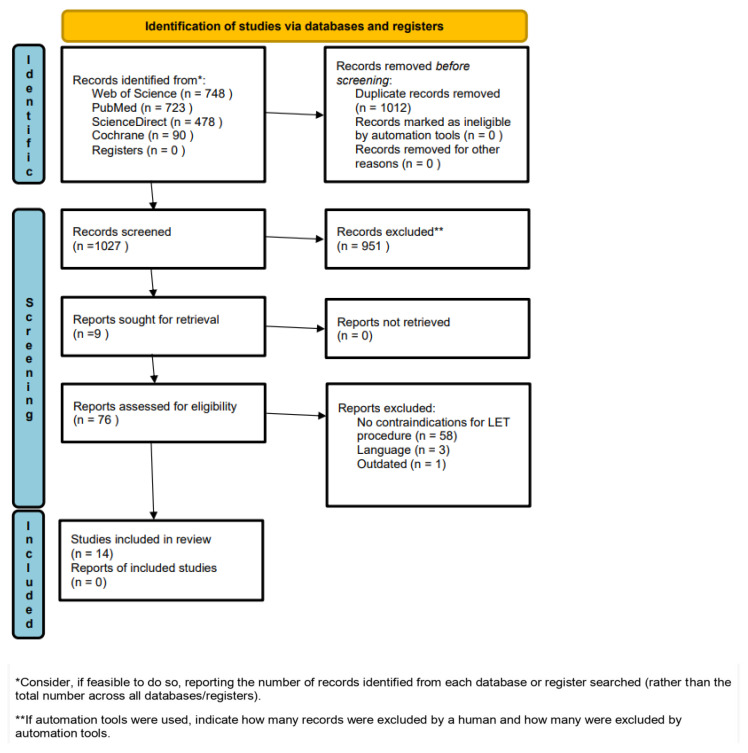
The PRISMA flow diagram.

This systematic review analyzed 14 articles published between 2012 and 2024 (Figure 1), with comprehensive demographic details summarized in Table 1. Geographically, the research primarily originated from Canada (*n* = 4), Italy (*n* = 3), and France (*n* = 3). The total cohort across all studies comprised 3452 subjects, including 1881 males and 1571 females. Notably, one study exclusively evaluated female participants [15]. The overall mean age was 23.2 years; for one study, age data was recalculated from subgroup averages to ensure consistency [16]. Methodologically, the inclusion consisted of eight retrospective and six prospective investigations, with Level III (*n* = 9) and Level I (*n* = 5) evidence being the most prevalent.

Subgroup analysis: We found that reporting consistency was directly linked to the study’s level of evidence. All Level I studies (*n* = 5) were in 100% agreement, identifying cartilage damage, multiligament injuries, and limb malalignment as primary contraindications. In contrast, Level III studies (*n* = 9) showed much more variety; for instance, malalignment was mentioned by only 22.2% of these authors, and cartilage damage by 55.6%. This highlights a much stronger consensus in high-quality trials compared to observational data.

## 4. Contraindications

This group of studies (*n* = 14) did not focus clearly on the LET procedure and described contraindications mainly in the context of ACLR and complex knee surgery. The most common contraindication to the LET surgery was articular cartilage damage (*n* = 10 studies) [1,20,21,22,23,24,25,27,29,31]. Additionally, in five studies, authors described the severity of the cartilage damage as symptomatic and requiring treatment beyond simple debridement [1,23,27,29,31]. Three additional authors specified symptomatic cartilage damage of grade ≥ 2 on the Outerbridge/ICRS scale as a contraindication [16,20,22]. The remaining authors utilized less precise terms.

Specifically, Perelli et al. identified cartilage lesions necessitating concurrent surgical management during ACLR as a contraindication, whereas Jacquet et al. employed more general terminology, referring to ‘bone or cartilage procedures’ as their exclusion criteria [20,21].

Concomitant ligamentous injury ranked as the second most frequent exclusion criterion, noted in nine studies. Within this classification, authors primarily cited multiligament instability—specifically cases involving the surgical repair of two or more ligaments—though the exact anatomical structures involved beyond the ACL were often left unspecified [1,20,21,23,26,27,28,29,30]. In two studies authors mentioned collateral ligament injury as a contraindication [22,24]. Additionally, Perelli S et al. specifically highlighted that the accompanying ligament injury (collateral ligaments and PCL) must be grade ≥ 2 [24].

However, all these authors included additional factors, besides the cartilage lesions and ligamentous injuries, as contraindications, presented in the text below.

Another commonly presented contraindication was knee malalignment in the frontal plane (*n* = 7 studies) [1,20,21,23,27,29,31]. However, six authors indicated that >3° of varus or valgus malalignment should be considered as a contraindication, while Viglietta et al. suggested a threshold of >10° [20,21,22,27,29,31]. Additionally, Hantouly et al. advised against performing LET in conjunction with a realignment osteotomy [31].

The remaining contraindications were presented less frequently and included categories related to the patient’s general health. Systemic diseases were identified as reasons for excluding patients in three studies [21,22,25]. Additionally, two authors focused on radiological signs of significant joint wear, specifically citing pre-operative Kellgren–Lawrence grade 3 or 4 osteoarthritis as a contraindication [16,22]. Other contraindications were mentioned only once, suggesting that they were of lesser significance.

It is worth mentioning that the study by Anderson et al., published in 1994, specified and focused on contraindications to the LET procedure [32]. They described associated ligament injuries, such as PCL or collateral ligament injuries, as reasons not to perform the LET procedure during complex surgery [32]. They also presented multiple-ligament injuries as a contraindication to performing the LET procedure. However, it was excluded from this systematic review due to the following reasons. It reports long-term follow-up outcomes of procedures performed in the 1980s. Although the authors present results of hamstring tendon ACLRs combined with Losee LETs, it is important to interpret the reported contraindications within the historical context of surgical practice at that time. During that period, the majority of ACLRs were frequently followed by prolonged immobilization in flexion casts. This type of rehabilitation is linked to a higher risk of joint stiffness, permanent loss of motion, and problems caused by over-tightening the knee [33]. Consequently, the negative outcomes and contraindications reported in this study may be more reflective of outdated surgical techniques and delayed rehabilitation strategies rather than inherent limitations of LET procedures themselves. All papers mentioning LET contraindications are listed in Table 2.

## 5. Discussion

Our systematic review was designed to identify and clarify the clinical reasons for avoiding LET during ACLR. Currently, LET is a vital technique for improving knee stability when used in tandem with ACLR. This research provides the first organized overview of contraindications for this procedure in the existing literature. Most authors in the selected papers aimed to achieve a uniform subject group for an experimental study. Consequently, they were unable to select specific and clear contraindications to LET modality, mixing the contraindications with ACLR and concurrent procedures commonly. Our study revealed that the unclear contraindications for the LET modality found in the available literature were articular cartilage damage (*n* = 10) and damage to another ligament in the knee, in addition to an ACL injury (*n* = 9). The absolute consensus among Level I studies suggests that cartilage damage, multiligament instability, and malalignment should be treated as the primary ‘red flags’ for LET. The higher variability seen in Level III studies likely reflects a less standardized approach to reporting in observational research rather than a lack of clinical importance.

The most commonly reported contraindication was articular cartilage damage. The complexity of this issue lies in the differing treatment algorithms depending on the degree of lesion, the various surgical techniques and the fact that an ideal protocol has not yet been provided [34]. Furthermore, consideration of that issue in this systematic review was also challenging, because most of the authors did not specify the degree of cartilage lesions and the type of applied treatment. Only a few researchers implemented the International Cartilage Repair Society (ICRS) scale, Outerbridge scale or Kellgren–Lawrence classification [22]. Furthermore, some authors only included their own criteria, such as a cartilage lesion requiring a procedure other than debridement or symptomatic chondral lesions without gradation [27,29,31,35]. However, based on the mentioned contraindications, it can be assumed that the authors primarily referred to cartilage damage of grade ≥ 2 on the Outerbridge/ICRS scale, which seems to represent reasonable advice, particularly considering that acute ACL injuries occur most commonly with mild chondral lesions, while high-grade defects range from 7 to 16% cases [36,37]. Severe knee arthritis and its radiographic evidence (Kellgren–Lawrence grade ≥ 3) were reported as unclear contraindications and they overlap with relative contraindications in isolated ACLR [38,39]. Performing isolated ACLR in moderate or severe knee arthritis may prevent secondary injuries to the cartilage and meniscus that accelerate degenerative changes and eventually may impact the time of total knee arthroplasty, especially in young, active patients [40]. The decision to include LET in ACLR for patients with cartilage defects remains a subject of debate. This controversy stems from the lack of certainty regarding whether the procedure speeds up the progression of knee osteoarthritis—an issue discussed in detail in the following section.

One of the most common contraindications was a multiligament injury, which refers to the simultaneous damage of one or more ligaments along with the ACL [1,20,21,22,23,24,26,27,28,29,30,31,41,42,43,44]. However, most of the studies did not clarify which particular ligaments were involved. When specific ligaments were mentioned, as in the study of Vadalà et al., who highlighted the collateral ligament tears, these lesions were classified under distinct categories: “concomitant injury of the internal or external collateral ligament” and “concomitant PCL insufficiency of the involved knee,” respectively [22]. In comparison, Perelli et al. identified severe damage (grade 2 or above) to the MCL, FCL, or PCL as specific reasons to avoid the LET procedure [24]. However, the situation becomes even more complex when the healing potential of ligaments is taken into account. The medial collateral ligament is generally considered to possess a strong capacity for healing, partly explained by the epiligament theory, which describes the epiligament as a connective tissue surrounding the ligament providing substrates necessary for the formation of cells, blood vessels, and extracellular matrix [45]. In contrast, the anterior cruciate ligament (ACL) demonstrates a limited healing capacity. This phenomenon has been attributed to the limited ability of cells and blood vessels to effectively bridge the ruptured ligament ends, as well as insufficient wound filling within the intra-articular environment. Moreover, the intra-articular ACL is exposed to synovial fluid, which inhibits fibroblast activity, while plasmin present in the synovial fluid degrades the fibrin clot, further compromising the healing process [46]. Nevertheless, it is believed that the biological and regenerative potential of the ACL is greater in its proximal portion than in the distal part. For this reason, primary repair of proximal ACL tears has been increasingly considered, as it allows preservation of the native ligament biology and may reduce the incidence of kinesiophobia and re-injury [47]. In any case, multiligament knee injuries present a challenge for orthopedic specialists and are associated with numerous issues. Complication rates after operative treatment in multiple-ligament injuries differ between studies due to many factors, especially timing of initial procedure (earlier than 3 weeks or later), that are being widely discussed [48]. However, complication rates range from 28% to 29.6% [49,50]. Commonly reported post-operative problems are: persistent instability, arthrofibrosis, hardware or suture complications, superficial or deep wound infections, and the necessity of revision surgery due to some mentioned issues [48,49,50,51,52]. The incidence of these complications is much higher compared to those observed in isolated ACLRs [50]. According to ACLR + LET modality, knowledge of the rate and types of complications in the present literature is limited. However, some short-term studies report that the ACLR + LET procedure may increase rates of hardware irritation, while medium-term research shows no increase in the complication rate [29,52]. Thus, considering this high rate of problems in operative management of multiple-ligament knee injuries, the addition of the LET procedure remains uncertain and may increase the incidence of complications. It is worth mentioning that PCL rupture, which constitutes a contraindication for the LET procedure, occurs with ACL injury in 48.2% cases and with posterolateral corner damage in 22.4% [53]. Thus, PCL lesions are mostly multiple-ligament knee injuries, and isolated injuries range from 15.3% to 26.4% [53,54].

Another consideration that is worth mentioning and supports multiple-ligament tears as a contraindication is the technical aspect of the LET procedure. There are various techniques for performing an LET procedure, but most require preparation of the iliotibial band strip and passing it under the FCL [55,56]. The prevalence of collateral ligament tears in patients who undergo ACLR is 19.2%, and in 12.3% of these subjects, there is a need for collateral ligament repair or reconstruction [57]. The same surgical technical issue relates to the posterolateral corner that consists of the FCL, the popliteus tendon and the popliteofibular ligament. Its rupture is commonly associated with ACL and PCL injuries, and only 28% of cases occur in isolation [58]. Thus, a concomitant FCL tear does not allow performance of an LET procedure, while medial collateral ligament damage (valgus laxity) with intact FCL may lead to a higher risk of over-constraint of the lateral knee compartment if LET is applied [29,56]. Subsequently, the lateral over-constraint may accelerate the development of osteoarthritis, but this phenomenon still remains a subject of debate [59]. For instance, some studies present the opposite, showing that LET does not contribute to the formation of degenerative changes [52,60,61,62]. It was also expanded on by Zaffagnini and colleagues, who showed that patients, averaging 24 years since undergoing ACLR with hamstring tendon and LET, did not develop lateral knee or patellofemoral osteoarthritis [63]. Similar conclusions were stated for ACLR + ALL reconstruction, with a mean follow-up of 104 months—there were no differences in joint space narrowing in the tibiofemoral compartment compared to isolated ACLR [64]. Furthermore, some studies demonstrate better radiographic and clinical outcomes for ACLR + LET compared to isolated ACLR, possibly due to its effect on the protection of meniscal function and repair [20,63,65]. It may be assumed that current long-term follow-up evidence does not consistently support an increased risk of knee osteoarthritis related to the addition of LET.

Interestingly, there was a contraindication regarding valgus or varus alignment of the knee. Most authors agreed that alignment of more than 3° was a contraindication. Viglietta et al. provided a notable exception, focusing on severe limb malalignment exceeding 10° of varus or valgus [20]. This is a critical factor, as substantial varus deformity is known to compromise ACL stability and is linked to a higher chance of graft failure due to the increased mechanical load placed on the ligament [66]. Additionally, research using MRI assessments has indicated that the LET graft may function as a load-sharing structure. By absorbing a portion of the mechanical stress acting on the knee, LET can decrease the burden on the ACL graft, which may ultimately support its biological healing and maturation [67]. However, in that study, limb alignments among patients were not specified, so it remains unknown what kind of forces an LET graft would absorb or generate in misalignments. The remaining contraindications that occur less frequently mainly overlap with relative contraindications for isolated ACLR [38,39].

Despite the large number of publications reviewed (1012 papers), precise contraindications to LET were difficult to establish. Of all the papers, only 14 passed through all stages of elimination. The relatively small amount of information on LET contraindications may suggest that this topic has not been well investigated yet. It may also indicate that the method is safe and applicable to a wide range of patients. However, further studies on contraindications to LET are necessary to be performed.

Our analysis has some limitations. First, the literature on this topic is limited and the majority of studies mix contraindications between the ACLR and LET procedures. Second, a significant number of papers provided ambiguous information regarding contraindications. In some cases, this ambiguity stemmed from the uniformity of the study group. In clinical research, a procedure is considered indicated when it is expected to provide a meaningful improvement in a specific medical condition, whereas it is considered non-indicated when its efficacy is unproven, and contraindicated when there is a risk of complications—potentially worsening the patient’s condition or, in the case of LET, leading to inferior outcomes compared with isolated ACLR. In the majority of the included studies, the reported criteria appear to reflect study-specific exclusion parameters rather than true clinical contraindications to LET. This distinction represents an important limitation in the interpretation of the available evidence, as inclusion and exclusion criteria are primarily designed to create homogeneous study populations and may not accurately define conditions under which the procedure should be avoided in routine clinical practice. Furthermore, our synthesis relied on a ‘vote-counting’ method, focusing on the frequency of reported contraindications across studies. As has been pointed out, a higher frequency of reporting does not necessarily correlate with the absolute strength of the underlying clinical evidence for a specific factor, but rather reflects current clinical consensus and common exclusion criteria in research protocols. Thirdly, a substantial number of the included studies provided Level III evidence. Additionally, a potential for selection bias was present, as our review was restricted to research published in the English language. Furthermore, one methodological limitation of the present systematic review is related to the assessment of the risk of bias. The classical Cochrane Risk of Bias tool was applied to evaluate the methodological quality of the included studies. However, this instrument was originally designed for randomized controlled trials, whereas the majority of studies included in this review were non-randomized in design. Therefore, the risk-of-bias evaluation should be interpreted with caution

## 6. Conclusions

This is the first study that systematized the contraindications for the LET procedure in ACLR. The unclear contraindications were numerous because the authors in the collected papers aimed to achieve a uniform subject group for an experimental study. Overall, the analyzed studies showed only a few contraindications specifically for the LET procedure. However, high heterogeneity among the studies highlights the need for additional high-quality studies, especially prospective and clearer definitions of contraindications to validate the findings.

## Figures and Tables

**Table 1:** Summary of methodological features and patient demographics from the literature evaluating contraindications for LET.

Author	Year	Country	Study Design	Level of Evidence	Type of Study	Number of Subjects	Male	Female	Mean Age (Years)
El-Azab et al. [20]	2023	Egypt, Austria	Randomized comparative study	1	Prospective	100	73	27	27.5
Viglietta et al. [21]	2021	Italy	Cohort study	3	Retrospective	164	126	38	27.3
Vadalà et al. [22]	2012	Italy	Comparative study	3	Prospective	60	0	60	27
Rezansoff et al. [23]	2023	Canada	Randomized controlled study	1	Prospective	553	265	288	18.8
Perelli et al. [24]	2022	Spain	Cohort study	3	Retrospective	66	43	23	13.65
Declercq et al. [16]	2022	France	Cohort study	3	Retrospective	86	69	17	25
Jacquet et al. [25]	2021	France	Cohort study	3	Retrospective	266	190	76	30.4
Monaco et al. [26]	2022	Italy	Cohort study	3	Retrospective	111	69	42	16.2
Getgood et al. [27]	2020	Canada	Randomized controlled trial	1	Prospective	618	297	321	18.9
Rowan et al. [28]	2019	UK	Propensity-matched case–control study	3	Prospective	273	154	119	31.6
Getgood and Moatshe et al. [1]	2020	Canada	Randomized controlled trial	1	Retrospective	356	154	202	18.9
Heard et al. [29]	2023	Canada	Randomized clinical trial	1	Prospective	618	302	316	18.9
Guy et al. [30]	2022	France	Cohort study	3	Retrospective	81	45	36	22.5
Hantouly et al. [31]	2023	Qatar	Cohort study	3	Retrospective	100	94	6	28.15

**Table 2:** Contraindications to LET procedure.

Author Contraindication	El-Azab et al. [20]	Viglietta et al. [21]	Vadalà et al. [22]	Rezansoff et al. [23]	Perelli et al. [24]	Declercq et al. [16]	Jacquet et al. [25]	Monaco et al. [26]	Getgood et al. [27]	Rowan et al. [28]	Getgood et al. [27]	Heard et al. [29]	Guy et al. [30]	Hantouly et al. [31]
Multiligament injury—two or more ligaments requiring surgical attention	yes	yes		yes				yes	yes	yes	yes	yes	yes	
Concomitant injury of the internal or the external collateral ligament			yes											
Concomitant grade 2 or higher tear of any other knee ligament (medial collateral ligament, lateral collateral ligament, posterior cruciate ligament)					yes									
Polytraumatized patient	yes													
Symptomatic articular cartilage defect requiring treatment, Outerbridge/ICRS > grade II	yes	yes	yes											
Symptomatic articular cartilage defect requiring treatment other than debridement				yes					yes		yes	yes		yes
Cartilage injuries requiring surgical treatment at the time of the ACL reconstruction					yes									
Pre-operative grade 3–4 chondropathy according to Kellgren–Lawrence classification						yes								
An associated bone or cartilage procedure							yes							
Concomitant realignment osteotomy														yes
>3° of varus or valgus malalignment	yes			yes					yes		yes	yes		yes
>10° of varus or valgus		yes												
Previous knee surgery: Total meniscectomy						yes								
Body mass index > 30		yes												
Age > 50 years at surgery		yes												
Development of post-operative septic arthritis		yes												
Presence of rheumatologic disorders		yes												
Concomitant systemic diseases			yes											
Chronic inflammatory joint disease							yes							
Pre-operative radiological signs of knee arthritis			yes											
Not participating in high-risk sports				yes										
Concomitant fractures (with the exception of Segond fractures)								yes						

## Data Availability

Data are unavailable due to privacy and ethical restrictions.

## Supplementary Materials

The following supporting information can be downloaded at: https://www.mdpi.com/article/10.3390/jcm15082821/s1.
