# Supplementary material for: Contraindications to Lateral Extra-Articular Tenodesis: A Systematic Review
Source: J Clin Med. 2026 Apr 8;15(8):2821. doi: 10.3390/jcm15082821 (PMC13116200; doi:10.3390/jcm15082821)
Supplement: Supplementary file 1 [file jcm-15-02821-s001.zip › jcm-4185827-supplementary.pdf]

OPEN ACCESS

Check for updates

# The PRISMA 2020 statement: an updated guideline for reporting systematic reviews

Matthew J Page,[1] Joanne E McKenzie,[1] Patrick M Bossuyt,[2] Isabelle Boutron,[3] Tammy C Hoffmann,[4] Cynthia D Mulrow,[5] Larissa Shamseer,[6] Jennifer M Tetzlaff,[7] Elie A Akl,[8] Sue E Brennan,[1] Roger Chou,[9] Julie Glanville,[10] Jeremy M Grimshaw,[11] Asbjørn Hróbjartsson,[12] Manoj M Lalu,[13] Tianjing Li,[14] Elizabeth W Loder,[15] Evan Mayo-Wilson,[16] Steve McDonald,[1] Luke A McGuinness,[17] Lesley A Stewart,[18] James Thomas,[19] Andrea C Tricco,[20] Vivian A Welch,[21] Penny Whiting,[17] David Moher[22]

BMJ: first published as 10.1136/bmj.n71 on 29 March 2021. Downloaded from https://www.bmj.com/ on 16 February 2026 by guest. Protected by copyright, including for uses related to text and data mining, AI training, and similar technologies.

For numbered affiliations see end of the article.

Correspondence to: M J Page matthew.page@monash.edu (ORCID 0000-0002-4242-7526)

Additional material is published online only. To view please visit the journal online.

Cite this as: *BMJ* 2021;372:n71
http://dx.doi.org/10.1136/bmj.n71

Accepted: 4 January 2021

The Preferred Reporting Items for Systematic reviews and Meta-Analyses (PRISMA) statement, published in 2009, was designed to help systematic reviewers transparently report why the review was done, what the authors did, and what they found. Over the past decade, advances in systematic review methodology and terminology have necessitated an update to the guideline. The PRISMA 2020 statement replaces the 2009 statement and includes new reporting guidance that reflects advances in methods to identify, select, appraise, and synthesise studies. The structure and presentation of the items have been modified to facilitate implementation. In this article, we present the PRISMA 2020 27-item checklist, an expanded checklist that details reporting recommendations for each item, the PRISMA 2020 abstract checklist, and the revised flow diagrams for original and updated reviews.

## SUMMARY POINTS

To ensure a systematic review is valuable to users, authors should prepare a transparent, complete, and accurate account of why the review was done, what they did, and what they found

The PRISMA 2020 statement provides updated reporting guidance for systematic reviews that reflects advances in methods to identify, select, appraise, and synthesise studies

The PRISMA 2020 statement consists of a 27-item checklist, an expanded checklist that details reporting recommendations for each item, the PRISMA 2020 abstract checklist, and revised flow diagrams for original and updated reviews

We anticipate that the PRISMA 2020 statement will benefit authors, editors, and peer reviewers of systematic reviews, and different users of reviews, including guideline developers, policy makers, healthcare providers, patients, and other stakeholders

Systematic reviews serve many critical roles. They can provide syntheses of the state of knowledge in a field, from which future research priorities can be identified; they can address questions that otherwise could not be answered by individual studies; they can identify problems in primary research that should be rectified in future studies; and they can generate or evaluate theories about how or why phenomena occur. Systematic reviews therefore generate various types of knowledge for different users of reviews (such as patients, healthcare providers, researchers, and policy makers).[1 2] To ensure a systematic review is valuable to users, authors should prepare a transparent, complete, and accurate account of why the review was done, what they did (such as how studies were identified and selected) and what they found (such as characteristics of contributing studies and results of meta-analyses). Up-to-date reporting guidance facilitates authors achieving this.[3]

The Preferred Reporting Items for Systematic reviews and Meta-Analyses (PRISMA) statement published in 2009 (hereafter referred to as PRISMA 2009)[4-10] is a reporting guideline designed to address poor reporting of systematic reviews.[11] The PRISMA 2009 statement comprised a checklist of 27 items recommended for reporting in systematic reviews and an "explanation and elaboration" paper[12-16] providing additional reporting guidance for each item, along with exemplars of reporting. The recommendations have been widely endorsed and adopted, as evidenced by its co-publication in multiple journals, citation in over 60 000 reports (Scopus, August 2020), endorsement from almost 200 journals and systematic review organisations, and adoption in various disciplines. Evidence from observational studies suggests that use of the PRISMA 2009 statement is associated with more complete reporting of systematic reviews,[17-20] although more could be done to improve adherence to the guideline.[21]

Many innovations in the conduct of systematic reviews have occurred since publication of the PRISMA 2009 statement. For example, technological advances have enabled the use of natural language processing and machine learning to identify relevant evidence,[22-24] methods have been proposed to

synthesise and present findings when meta-analysis is not possible or appropriate,[25-27] and new methods have been developed to assess the risk of bias in results of included studies.[28 29] Evidence on sources of bias in systematic reviews has accrued, culminating in the development of new tools to appraise the conduct of systematic reviews.[30 31] Terminology used to describe particular review processes has also evolved, as in the shift from assessing "quality" to assessing "certainty" in the body of evidence.[32] In addition, the publishing landscape has transformed, with multiple avenues now available for registering and disseminating systematic review protocols,[33 34] disseminating reports of systematic reviews, and sharing data and materials, such as preprint servers and publicly accessible repositories. To capture these advances in the reporting of systematic reviews necessitated an update to the PRISMA 2009 statement.

### Development of PRISMA 2020

A complete description of the methods used to develop PRISMA 2020 is available elsewhere.[35] We identified PRISMA 2009 items that were often reported incompletely by examining the results of studies investigating the transparency of reporting of published reviews.[17 21 36 37] We identified possible modifications to the PRISMA 2009 statement by reviewing 60 documents providing reporting guidance for systematic reviews (including reporting guidelines, handbooks, tools, and meta-research studies).[38] These reviews of the literature were used to inform the content of a survey with suggested possible modifications to the 27 items in PRISMA 2009 and possible additional items. Respondents were asked whether they believed we should keep each PRISMA 2009 item as is, modify it, or remove it, and whether we should add each additional item. Systematic review methodologists and journal editors were invited to complete the online survey (110 of 220 invited responded). We discussed proposed content and wording of the PRISMA 2020 statement, as informed by the review and survey results, at a 21-member, two-day, in-person meeting in September 2018 in Edinburgh, Scotland. Throughout 2019 and 2020, we circulated an initial draft and five revisions of the checklist and explanation and elaboration paper to co-authors for feedback. In April 2020, we invited 22 systematic reviewers who had expressed interest in providing feedback on the PRISMA 2020 checklist to share their views (via an online survey) on the layout and terminology used in a preliminary version of the checklist. Feedback was received from 15 individuals and considered by the first author, and any revisions deemed necessary were incorporated before the final version was approved and endorsed by all co-authors.

### The PRISMA 2020 statement

#### Scope of the guideline

The PRISMA 2020 statement has been designed primarily for systematic reviews of studies that evaluate the effects of health interventions, irrespective of the design of the included studies. However, the checklist items are applicable to reports of systematic reviews evaluating other interventions (such as social or educational interventions), and many items are applicable to systematic reviews with objectives other than evaluating interventions (such as evaluating aetiology, prevalence, or prognosis). PRISMA 2020 is intended for use in systematic reviews that include synthesis (such as pairwise meta-analysis or other statistical synthesis methods) or do not include synthesis (for example, because only one eligible study is identified). The PRISMA 2020 items are relevant for mixed-methods systematic reviews (which include quantitative and qualitative studies), but reporting guidelines addressing the presentation and synthesis of qualitative data should also be consulted.[39 40] PRISMA 2020 can be used for original systematic reviews, updated systematic reviews, or continually updated ("living") systematic reviews. However, for updated and living systematic reviews, there may be some additional considerations that need to be addressed. Where there is relevant content from other reporting guidelines, we reference these guidelines within the items in the explanation and elaboration paper[41] (such as PRISMA-Search[42] in items 6 and 7, Synthesis without meta-analysis (SWiM) reporting guideline[27] in item 13d). Box 1 includes a glossary of terms used throughout the PRISMA 2020 statement.

PRISMA 2020 is not intended to guide systematic review conduct, for which comprehensive resources are available.[43-46] However, familiarity with PRISMA 2020 is useful when planning and conducting systematic reviews to ensure that all recommended information is captured. PRISMA 2020 should not be used to assess the conduct or methodological quality of systematic reviews; other tools exist for this purpose.[30 31] Furthermore, PRISMA 2020 is not intended to inform the reporting of systematic review protocols, for which a separate statement is available (PRISMA for Protocols (PRISMA-P) 2015 statement[47 48]). Finally, extensions to the PRISMA 2009 statement have been developed to guide reporting of network meta-analyses,[49] meta-analyses of individual participant data,[50] systematic reviews of harms,[51] systematic reviews of diagnostic test accuracy studies,[52] and scoping reviews[53]; for these types of reviews we recommend authors report their review in accordance with the recommendations in PRISMA 2020 along with the guidance specific to the extension.

#### How to use PRISMA 2020

The PRISMA 2020 statement (including the checklists, explanation and elaboration, and flow diagram) replaces the PRISMA 2009 statement, which should no longer be used. Box 2 summarises noteworthy changes from the PRISMA 2009 statement. The PRISMA 2020 checklist includes seven sections with 27 items, some of which include sub-items (table 1). A checklist for journal and conference abstracts for systematic reviews is included in PRISMA 2020. This abstract checklist is an update of the 2013 PRISMA for Abstracts statement,[54] reflecting new and modified

BMJ: first published as 10.1136/bmj.n71 on 29 March 2021. Downloaded from https://www.bmj.com/ on 16 February 2026 by guest. Protected by copyright, including for uses related to text and data mining, AI training, and similar technologies.

**Box 1: Glossary of terms**

- *Systematic review*—A review that uses explicit, systematic methods to collate and synthesise findings of studies that address a clearly formulated question[43]
- *Statistical synthesis*—The combination of quantitative results of two or more studies. This encompasses meta-analysis of effect estimates (described below) and other methods, such as combining P values, calculating the range and distribution of observed effects, and vote counting based on the direction of effect (see McKenzie and Brennan[25] for a description of each method)
- *Meta-analysis of effect estimates*—A statistical technique used to synthesise results when study effect estimates and their variances are available, yielding a quantitative summary of results[25]
- *Outcome*—An event or measurement collected for participants in a study (such as quality of life, mortality)
- *Result*—The combination of a point estimate (such as a mean difference, risk ratio, or proportion) and a measure of its precision (such as a confidence/credible interval) for a particular outcome
- *Report*—A document (paper or electronic) supplying information about a particular study. It could be a journal article, preprint, conference abstract, study register entry, clinical study report, dissertation, unpublished manuscript, government report, or any other document providing relevant information
- *Record*—The title or abstract (or both) of a report indexed in a database or website (such as a title or abstract for an article indexed in Medline). Records that refer to the same report (such as the same journal article) are "duplicates"; however, records that refer to reports that are merely similar (such as a similar abstract submitted to two different conferences) should be considered unique.
- *Study*—An investigation, such as a clinical trial, that includes a defined group of participants and one or more interventions and outcomes. A "study" might have multiple reports. For example, reports could include the protocol, statistical analysis plan, baseline characteristics, results for the primary outcome, results for harms, results for secondary outcomes, and results for additional mediator and moderator analyses

content in PRISMA 2020 (table 2). A template PRISMA flow diagram is provided, which can be modified depending on whether the systematic review is original or updated (fig 1).

We recommend authors refer to PRISMA 2020 early in the writing process, because prospective consideration of the items may help to ensure that all the items are addressed. To help keep track of which items have been reported, the PRISMA statement website (http://www.prisma-statement.org/) includes fillable templates of the checklists to download and complete (also available in the data supplement on bmj.com). We have also created a web application that allows users to complete the checklist via a user-friendly interface[58] (available at https://prisma.shinyapps.io/checklist/ and adapted from the Transparency Checklist app[59]). The completed checklist can be exported to Word or PDF. Editable templates of the flow diagram can also be downloaded from the PRISMA statement website.

We have prepared an updated explanation and elaboration paper, in which we explain why reporting of each item is recommended and present bullet points that detail the reporting recommendations (which we

**Box 2: Noteworthy changes to the PRISMA 2009 statement**

- Inclusion of the abstract reporting checklist within PRISMA 2020 (see item #2 and table 2).
- Movement of the 'Protocol and registration' item from the start of the Methods section of the checklist to a new Other section, with addition of a sub-item recommending authors describe amendments to information provided at registration or in the protocol (see item #24a-24c).
- Modification of the 'Search' item to recommend authors present full search strategies for *all* databases, registers and websites searched, not just at least one database (see item #7).
- Modification of the 'Study selection' item in the Methods section to emphasise the reporting of how many reviewers screened each record and each report retrieved, whether they worked independently, and if applicable, details of automation tools used in the process (see item #8).
- Addition of a sub-item to the 'Data items' item recommending authors report how outcomes were defined, which results were sought, and methods for selecting a subset of results from included studies (see item #10a).
- Splitting of the 'Synthesis of results' item in the Methods section into six sub-items recommending authors describe: the processes used to decide which studies were eligible for each synthesis; any methods required to prepare the data for synthesis; any methods used to tabulate or visually display results of individual studies and syntheses; any methods used to synthesise results; any methods used to explore possible causes of heterogeneity among study results (such as subgroup analysis, meta-regression); and any sensitivity analyses used to assess robustness of the synthesised results (see item #13a-13f).
- Addition of a sub-item to the 'Study selection' item in the Results section recommending authors cite studies that might appear to meet the inclusion criteria, but which were excluded, and explain why they were excluded (see item #16b).
- Splitting of the 'Synthesis of results' item in the Results section into four sub-items recommending authors: briefly summarise the characteristics and risk of bias among studies contributing to the synthesis; present results of all statistical syntheses conducted; present results of any investigations of possible causes of heterogeneity among study results; and present results of any sensitivity analyses (see item #20a-20d).
- Addition of new items recommending authors report methods for and results of an assessment of certainty (or confidence) in the body of evidence for an outcome (see items #15 and #22).
- Addition of a new item recommending authors declare any competing interests (see item #26).
- Addition of a new item recommending authors indicate whether data, analytic code and other materials used in the review are publicly available and if so, where they can be found (see item #27).

BMJ: first published as 10.1136/bmj.n71 on 29 March 2021. Downloaded from https://www.bmj.com/ on 16 February 2026 by guest. Protected by copyright, including for uses related to text and data mining, AI training, and similar technologies.

Table 1 | PRISMA 2020 item checklist

| Section and topic | Item # | Checklist item | Location where item is reported |
|---|---|---|---|
| **Title** | | | |
| Title | 1 | Identify the report as a systematic review. | |
| **Abstract** | | | |
| Abstract | 2 | See the PRISMA 2020 for Abstracts checklist (table 2). | |
| **Introduction** | | | |
| Rationale | 3 | Describe the rationale for the review in the context of existing knowledge. | |
| Objectives | 4 | Provide an explicit statement of the objective(s) or question(s) the review addresses. | |
| **Methods** | | | |
| Eligibility criteria | 5 | Specify the inclusion and exclusion criteria for the review and how studies were grouped for the syntheses. | |
| Information sources | 6 | Specify all databases, registers, websites, organisations, reference lists and other sources searched or consulted to identify studies. Specify the date when each source was last searched or consulted. | |
| Search strategy | 7 | Present the full search strategies for all databases, registers and websites, including any filters and limits used. | |
| Selection process | 8 | Specify the methods used to decide whether a study met the inclusion criteria of the review, including how many reviewers screened each record and each report retrieved, whether they worked independently, and if applicable, details of automation tools used in the process. | |
| Data collection process | 9 | Specify the methods used to collect data from reports, including how many reviewers collected data from each report, whether they worked independently, any processes for obtaining or confirming data from study investigators, and if applicable, details of automation tools used in the process. | |
| Data items | 10a | List and define all outcomes for which data were sought. Specify whether all results that were compatible with each outcome domain in each study were sought (e.g. for all measures, time points, analyses), and if not, the methods used to decide which results to collect. | |
| | 10b | List and define all other variables for which data were sought (e.g. participant and intervention characteristics, funding sources). Describe any assumptions made about any missing or unclear information. | |
| Study risk of bias assessment | 11 | Specify the methods used to assess risk of bias in the included studies, including details of the tool(s) used, how many reviewers assessed each study and whether they worked independently, and if applicable, details of automation tools used in the process. | |
| Effect measures | 12 | Specify for each outcome the effect measure(s) (e.g. risk ratio, mean difference) used in the synthesis or presentation of results. | |
| Synthesis methods | 13a | Describe the processes used to decide which studies were eligible for each synthesis (e.g. tabulating the study intervention characteristics and comparing against the planned groups for each synthesis (item #5)). | |
| | 13b | Describe any methods required to prepare the data for presentation or synthesis, such as handling of missing summary statistics, or data conversions. | |
| | 13c | Describe any methods used to tabulate or visually display results of individual studies and syntheses. | |
| | 13d | Describe any methods used to synthesise results and provide a rationale for the choice(s). If meta-analysis was performed, describe the model(s), method(s) to identify the presence and extent of statistical heterogeneity, and software package(s) used. | |
| | 13e | Describe any methods used to explore possible causes of heterogeneity among study results (e.g. subgroup analysis, meta-regression). | |
| | 13f | Describe any sensitivity analyses conducted to assess robustness of the synthesised results. | |
| Reporting bias assessment | 14 | Describe any methods used to assess risk of bias due to missing results in a synthesis (arising from reporting biases). | |
| Certainty assessment | 15 | Describe any methods used to assess certainty (or confidence) in the body of evidence for an outcome. | |
| **Results** | | | |
| Study selection | 16a | Describe the results of the search and selection process, from the number of records identified in the search to the number of studies included in the review, ideally using a flow diagram (see fig 1). | |
| | 16b | Cite studies that might appear to meet the inclusion criteria, but which were excluded, and explain why they were excluded. | |
| Study characteristics | 17 | Cite each included study and present its characteristics. | |
| Risk of bias in studies | 18 | Present assessments of risk of bias for each included study. | |
| Results of individual studies | 19 | For all outcomes, present, for each study: (a) summary statistics for each group (where appropriate) and (b) an effect estimate and its precision (e.g. confidence/credible interval), ideally using structured tables or plots. | |
| Results of syntheses | 20a | For each synthesis, briefly summarise the characteristics and risk of bias among contributing studies. | |
| | 20b | Present results of all statistical syntheses conducted. If meta-analysis was done, present for each the summary estimate and its precision (e.g. confidence/credible interval) and measures of statistical heterogeneity. If comparing groups, describe the direction of the effect. | |
| | 20c | Present results of all investigations of possible causes of heterogeneity among study results. | |
| | 20d | Present results of all sensitivity analyses conducted to assess the robustness of the synthesised results. | |
| Reporting biases | 21 | Present assessments of risk of bias due to missing results (arising from reporting biases) for each synthesis assessed. | |
| Certainty of evidence | 22 | Present assessments of certainty (or confidence) in the body of evidence for each outcome assessed. | |
| **Discussion** | | | |
| Discussion | 23a | Provide a general interpretation of the results in the context of other evidence. | |
| | 23b | Discuss any limitations of the evidence included in the review. | |
| | 23c | Discuss any limitations of the review processes used. | |
| | 23d | Discuss implications of the results for practice, policy, and future research. | |
| **Other information** | | | |
| Registration and protocol | 24a | Provide registration information for the review, including register name and registration number, or state that the review was not registered. | |
| | 24b | Indicate where the review protocol can be accessed, or state that a protocol was not prepared. | |
| | 24c | Describe and explain any amendments to information provided at registration or in the protocol. | |
| Support | 25 | Describe sources of financial or non-financial support for the review, and the role of the funders or sponsors in the review. | |
| Competing interests | 26 | Declare any competing interests of review authors. | |
| Availability of data, code, and other materials | 27 | Report which of the following are publicly available and where they can be found: template data collection forms; data extracted from included studies; data used for all analyses; analytic code; any other materials used in the review. | |

BMJ: first published as 10.1136/bmj.n71 on 29 March 2021. Downloaded from https://www.bmj.com/ on 16 February 2026 by guest. Protected by copyright, including for uses related to text and data mining, AI training, and similar technologies.

Table 2 | PRISMA 2020 for Abstracts checklist*

| Section and topic | Item # | Checklist item |
|---|---|---|
| **Title** | | |
| Title | 1 | Identify the report as a systematic review. |
| **Background** | | |
| Objectives | 2 | Provide an explicit statement of the main objective(s) or question(s) the review addresses. |
| **Methods** | | |
| Eligibility criteria | 3 | Specify the inclusion and exclusion criteria for the review. |
| Information sources | 4 | Specify the information sources (e.g. databases, registers) used to identify studies and the date when each was last searched. |
| Risk of bias | 5 | Specify the methods used to assess risk of bias in the included studies. |
| Synthesis of results | 6 | Specify the methods used to present and synthesise results. |
| **Results** | | |
| Included studies | 7 | Give the total number of included studies and participants and summarise relevant characteristics of studies. |
| Synthesis of results | 8 | Present results for main outcomes, preferably indicating the number of included studies and participants for each. If meta-analysis was done, report the summary estimate and confidence/credible interval. If comparing groups, indicate the direction of the effect (i.e. which group is favoured). |
| **Discussion** | | |
| Limitations of evidence | 9 | Provide a brief summary of the limitations of the evidence included in the review (e.g. study risk of bias, inconsistency and imprecision). |
| Interpretation | 10 | Provide a general interpretation of the results and important implications. |
| **Other** | | |
| Funding | 11 | Specify the primary source of funding for the review. |
| Registration | 12 | Provide the register name and registration number. |

*This abstract checklist retains the same items as those included in the PRISMA for Abstracts statement published in 2013,[54] but has been revised to make the wording consistent with the PRISMA 2020 statement and includes a new item recommending authors specify the methods used to present and synthesise results (item #6).

refer to as elements).[41] The bullet-point structure is new to PRISMA 2020 and has been adopted to facilitate implementation of the guidance.[60 61] An expanded checklist, which comprises an abridged version of the elements presented in the explanation and elaboration paper, with references and some examples removed, is available in the data supplement on bmj.com. Consulting the explanation and elaboration paper is recommended if further clarity or information is required.

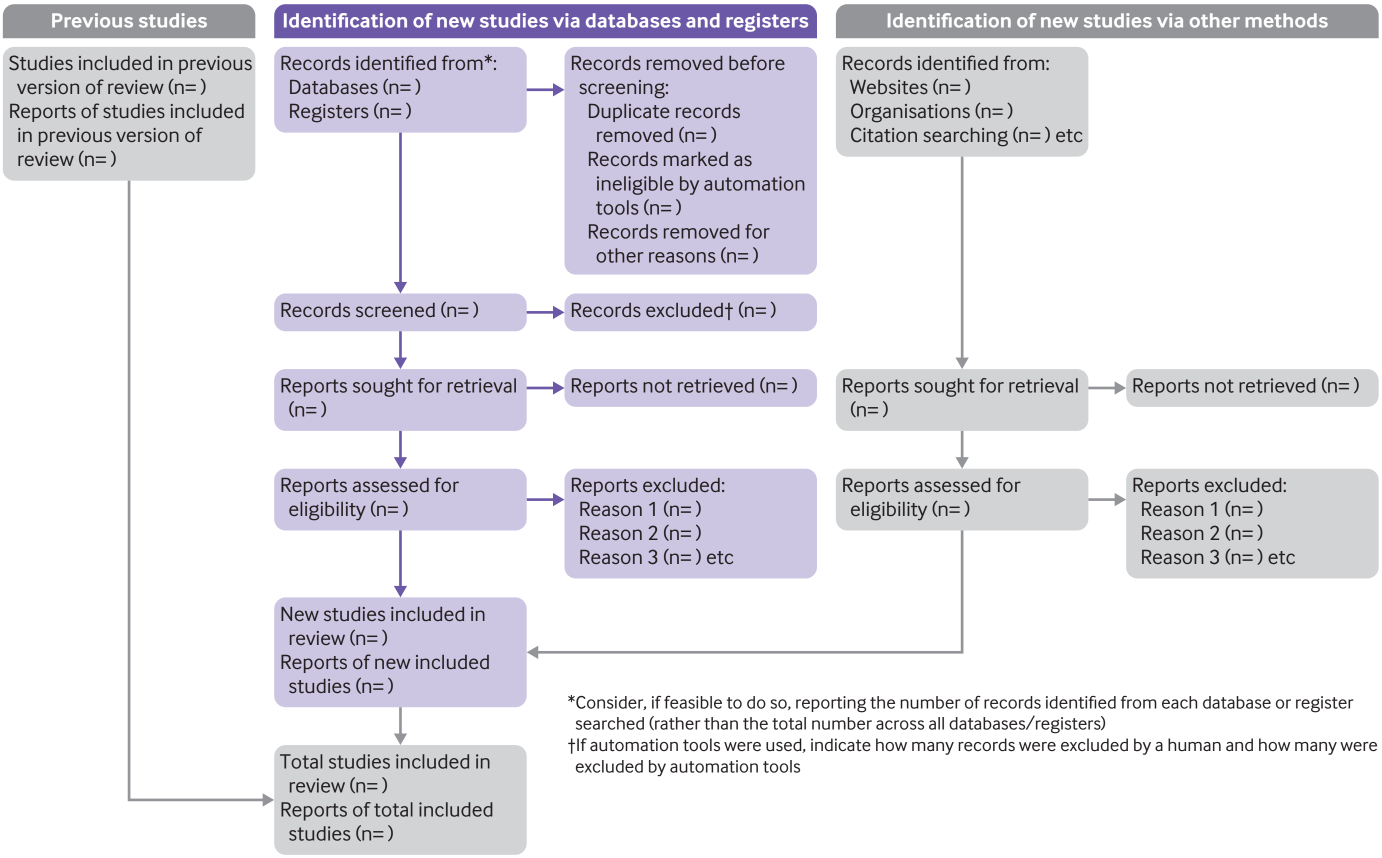


Fig 1 | PRISMA 2020 flow diagram template for systematic reviews. The new design is adapted from flow diagrams proposed by Boers,[55] Mayo-Wilson et al.[56] and Stovold et al.[57] The boxes in grey should only be completed if applicable; otherwise they should be removed from the flow diagram. Note that a "report" could be a journal article, preprint, conference abstract, study register entry, clinical study report, dissertation, unpublished manuscript, government report or any other document providing relevant information.

BMJ: first published as 10.1136/bmj.n71 on 29 March 2021. Downloaded from https://www.bmj.com/ on 16 February 2026 by guest. Protected by copyright, including for uses related to text and data mining, AI training, and similar technologies.

Journals and publishers might impose word and section limits, and limits on the number of tables and figures allowed in the main report. In such cases, if the relevant information for some items already appears in a publicly accessible review protocol, referring to the protocol may suffice. Alternatively, placing detailed descriptions of the methods used or additional results (such as for less critical outcomes) in supplementary files is recommended. Ideally, supplementary files should be deposited to a general-purpose or institutional open-access repository that provides free and permanent access to the material (such as Open Science Framework, Dryad, figshare). A reference or link to the additional information should be included in the main report. Finally, although PRISMA 2020 provides a template for where information might be located, the suggested location should not be seen as prescriptive; the guiding principle is to ensure the information is reported.

## Discussion

Use of PRISMA 2020 has the potential to benefit many stakeholders. Complete reporting allows readers to assess the appropriateness of the methods, and therefore the trustworthiness of the findings. Presenting and summarising characteristics of studies contributing to a synthesis allows healthcare providers and policy makers to evaluate the applicability of the findings to their setting. Describing the certainty in the body of evidence for an outcome and the implications of findings should help policy makers, managers, and other decision makers formulate appropriate recommendations for practice or policy. Complete reporting of all PRISMA 2020 items also facilitates replication and review updates, as well as inclusion of systematic reviews in overviews (of systematic reviews) and guidelines, so teams can leverage work that is already done and decrease research waste.[36 62 63]

We updated the PRISMA 2009 statement by adapting the EQUATOR Network's guidance for developing health research reporting guidelines.[64] We evaluated the reporting completeness of published systematic reviews,[17 21 36 37] reviewed the items included in other documents providing guidance for systematic reviews,[38] surveyed systematic review methodologists and journal editors for their views on how to revise the original PRISMA statement,[35] discussed the findings at an in-person meeting, and prepared this document through an iterative process. Our recommendations are informed by the reviews and survey conducted before the in-person meeting, theoretical considerations about which items facilitate replication and help users assess the risk of bias and applicability of systematic reviews, and co-authors' experience with authoring and using systematic reviews.

Various strategies to increase the use of reporting guidelines and improve reporting have been proposed. They include educators introducing reporting guidelines into graduate curricula to promote good reporting habits of early career scientists[65]; journal editors and regulators endorsing use of reporting guidelines[18]; peer reviewers evaluating adherence to reporting guidelines[61 66]; journals requiring authors to indicate where in their manuscript they have adhered to each reporting item[67]; and authors using online writing tools that prompt complete reporting at the writing stage.[60] Multi-pronged interventions, where more than one of these strategies are combined, may be more effective (such as completion of checklists coupled with editorial checks).[68] However, of 31 interventions proposed to increase adherence to reporting guidelines, the effects of only 11 have been evaluated, mostly in observational studies at high risk of bias due to confounding.[69] It is therefore unclear which strategies should be used. Future research might explore barriers and facilitators to the use of PRISMA 2020 by authors, editors, and peer reviewers, designing interventions that address the identified barriers, and evaluating those interventions using randomised trials. To inform possible revisions to the guideline, it would also be valuable to conduct think-aloud studies[70] to understand how systematic reviewers interpret the items, and reliability studies to identify items where there is varied interpretation of the items.

We encourage readers to submit evidence that informs any of the recommendations in PRISMA 2020 (via the PRISMA statement website: http://www.prisma-statement.org/). To enhance accessibility of PRISMA 2020, several translations of the guideline are under way (see available translations at the PRISMA statement website). We encourage journal editors and publishers to raise awareness of PRISMA 2020 (for example, by referring to it in journal "Instructions to authors"), endorsing its use, advising editors and peer reviewers to evaluate submitted systematic reviews against the PRISMA 2020 checklists, and making changes to journal policies to accommodate the new reporting recommendations. We recommend existing PRISMA extensions[47 49 50 51-53 71 72] be updated to reflect PRISMA 2020 and advise developers of new PRISMA extensions to use PRISMA 2020 as the foundation document.

## Conclusion

We anticipate that the PRISMA 2020 statement will benefit authors, editors, and peer reviewers of systematic reviews, and different users of reviews, including guideline developers, policy makers, healthcare providers, patients, and other stakeholders. Ultimately, we hope that uptake of the guideline will lead to more transparent, complete, and accurate reporting of systematic reviews, thus facilitating evidence based decision making.

### Author affiliations

[1]School of Public Health and Preventive Medicine, Monash University, Melbourne, Australia

[2]Department of Clinical Epidemiology, Biostatistics and Bioinformatics, Amsterdam University Medical Centres, University of Amsterdam, Amsterdam, Netherlands

[3]Université de Paris, Centre of Epidemiology and Statistics (CRESS), Inserm, F 75004 Paris, France

[4]Institute for Evidence-Based Healthcare, Faculty of Health Sciences and Medicine, Bond University, Gold Coast, Australia

BMJ: first published as 10.1136/bmj.n71 on 29 March 2021. Downloaded from https://www.bmj.com/ on 16 February 2026 by guest. Protected by copyright, including for uses related to text and data mining, AI training, and similar technologies.

[5]University of Texas Health Science Center at San Antonio, San Antonio, Texas, USA; *Annals of Internal Medicine*

[6]Knowledge Translation Program, Li Ka Shing Knowledge Institute, Toronto, Canada; School of Epidemiology and Public Health, Faculty of Medicine, University of Ottawa, Ottawa, Canada

[7]Evidence Partners, Ottawa, Canada

[8]Clinical Research Institute, American University of Beirut, Beirut, Lebanon; Department of Health Research Methods, Evidence, and Impact, McMaster University, Hamilton, Ontario, Canada

[9]Department of Medical Informatics and Clinical Epidemiology, Oregon Health & Science University, Portland, Oregon, USA

[10]York Health Economics Consortium (YHEC Ltd), University of York, York, UK

[11]Clinical Epidemiology Program, Ottawa Hospital Research Institute, Ottawa, Canada; School of Epidemiology and Public Health, University of Ottawa, Ottawa, Canada; Department of Medicine, University of Ottawa, Ottawa, Canada

[12]Centre for Evidence-Based Medicine Odense (CEBMO) and Cochrane Denmark, Department of Clinical Research, University of Southern Denmark, Odense, Denmark; Open Patient data Exploratory Network (OPEN), Odense University Hospital, Odense, Denmark

[13]Department of Anesthesiology and Pain Medicine, The Ottawa Hospital, Ottawa, Canada; Clinical Epidemiology Program, Blueprint Translational Research Group, Ottawa Hospital Research Institute, Ottawa, Canada; Regenerative Medicine Program, Ottawa Hospital Research Institute, Ottawa, Canada

[14]Department of Ophthalmology, School of Medicine, University of Colorado Denver, Denver, Colorado, United States; Department of Epidemiology, Johns Hopkins Bloomberg School of Public Health, Baltimore, Maryland, USA

[15]Division of Headache, Department of Neurology, Brigham and Women's Hospital, Harvard Medical School, Boston, Massachusetts, USA; Head of Research, *The BMJ*, London, UK

[16]Department of Epidemiology and Biostatistics, Indiana University School of Public Health-Bloomington, Bloomington, Indiana, USA

[17]Population Health Sciences, Bristol Medical School, University of Bristol, Bristol, UK

[18]Centre for Reviews and Dissemination, University of York, York, UK

[19]EPPI-Centre, UCL Social Research Institute, University College London, London, UK

[20]Li Ka Shing Knowledge Institute of St. Michael's Hospital, Unity Health Toronto, Toronto, Canada; Epidemiology Division of the Dalla Lana School of Public Health and the Institute of Health Management, Policy, and Evaluation, University of Toronto, Toronto, Canada; Queen's Collaboration for Health Care Quality Joanna Briggs Institute Centre of Excellence, Queen's University, Kingston, Canada

[21]Methods Centre, Bruyère Research Institute, Ottawa, Ontario, Canada; School of Epidemiology and Public Health, Faculty of Medicine, University of Ottawa, Ottawa, Canada

[22]Centre for Journalology, Clinical Epidemiology Program, Ottawa Hospital Research Institute, Ottawa, Canada; School of Epidemiology and Public Health, Faculty of Medicine, University of Ottawa, Ottawa, Canada

We dedicate this paper to the late Douglas G Altman and Alessandro Liberati, whose contributions were fundamental to the development and implementation of the original PRISMA statement.

We thank the following contributors who completed the survey to inform discussions at the development meeting: Xavier Armoiry, Edoardo Aromataris, Ana Patricia Ayala, Ethan M Balk, Virginia Barbour, Elaine Beller, Jesse A Berlin, Lisa Bero, Zhao-Xiang Bian, Jean Joel Bigna, Ferrán Catalá-López, Anna Chaimani, Mike Clarke, Tammy Clifford, Ioana A Cristea, Miranda Cumpston, Sofia Dias, Corinna Dressler, Ivan D Florez, Joel J Gagnier, Chantelle Garritty, Long Ge, Davina Ghersi, Sean Grant, Gordon Guyatt, Neal R Haddaway, Julian PT Higgins, Sally Hopewell, Brian Hutton, Jamie J Kirkham, Jos Kleijnen, Julia Koricheva, Joey SW Kwong, Toby J Lasserson, Julia H Littell, Yoon K Loke, Malcolm R Macleod, Chris G Maher, Ana Marušic, Dimitris Mavridis, Jessie McGowan, Matthew DF McInnes, Philippa Middleton, Karel G Moons, Zachary Munn, Jane Noyes, Barbara Nußbaumer-Streit, Donald L Patrick, Tatiana Pereira-Cenci, Ba' Pham, Bob Phillips, Dawid Pieper, Michelle Pollock, Daniel S Quintana, Drummond Rennie, Melissa L Rethlefsen, Hannah R Rothstein, Maroeska M Rovers, Rebecca Ryan, Georgia Salanti, Ian J Saldanha, Margaret Sampson, Nancy Santesso, Rafael Sarkis-Onofre, Jelena Savović, Christopher H Schmid, Kenneth F Schulz, Guido Schwarzer, Beverley J Shea, Paul G Shekelle, Farhad Shokraneh, Mark Simmonds, Nicole Skoetz, Sharon E Straus, Anneliese Synnot, Emily E Tanner-Smith, Brett D Thombs, Hilary Thomson, Alexander Tsertsvadze, Peter Tugwell, Tari Turner, Lesley Uttley, Jeffrey C Valentine, Matt Vassar, Areti Angeliki Veroniki, Meera Viswanathan, Cole Wayant, Paul Whaley, and Kehu Yang. We thank the following contributors who provided feedback on a preliminary version of the PRISMA 2020 checklist: Jo Abbott, Fionn Büttner, Patricia Correia-Santos, Victoria Freeman, Emily A Hennessy, Rakibul Islam, Amalia (Emily) Karahalios, Kasper Krommes, Andreas Lundh, Dafne Port Nascimento, Davina Robson, Catherine Schenck-Yglesias, Mary M Scott, Sarah Tanveer and Pavel Zhelnov. We thank Abigail H Goben, Melissa L Rethlefsen, Tanja Rombey, Anna Scott, and Farhad Shokraneh for their helpful comments on the preprints of the PRISMA 2020 papers. We thank Edoardo Aromataris, Stephanie Chang, Toby Lasserson and David Schriger for their helpful peer review comments on the PRISMA 2020 papers.

Contributors: JEM and DM are joint senior authors. MJP, JEM, PMB, IB, TCH, CDM, LS, and DM conceived this paper and designed the literature review and survey conducted to inform the guideline content. MJP conducted the literature review, administered the survey and analysed the data for both. MJP prepared all materials for the development meeting. MJP and JEM presented proposals at the development meeting. All authors except for TCH, JMT, EAA, SEB, and LAM attended the development meeting. MJP and JEM took and consolidated notes from the development meeting. MJP and JEM led the drafting and editing of the article. JEM, PMB, IB, TCH, LS, JMT, EAA, SEB, RC, JG, AH, TL, EMW, SM, LAM, LAS, JT, ACT, PW, and DM drafted particular sections of the article. All authors were involved in revising the article critically for important intellectual content. All authors approved the final version of the article. MJP is the guarantor of this work. The corresponding author attests that all listed authors meet authorship criteria and that no others meeting the criteria have been omitted.

Funding: There was no direct funding for this research. MJP is supported by an Australian Research Council Discovery Early Career Researcher Award (DE200101618) and was previously supported by an Australian National Health and Medical Research Council (NHMRC) Early Career Fellowship (1088535) during the conduct of this research. JEM is supported by an Australian NHMRC Career Development Fellowship (1143429). TCH is supported by an Australian NHMRC Senior Research Fellowship (1154607). JMT is supported by Evidence Partners Inc. JMG is supported by a Tier 1 Canada Research Chair in Health Knowledge Transfer and Uptake. MML is supported by The Ottawa Hospital Anaesthesia Alternate Funds Association and a Faculty of Medicine Junior Research Chair. TL is supported by funding from the National Eye Institute (UG1EY020522), National Institutes of Health, United States. LAM is supported by a National Institute for Health Research Doctoral Research Fellowship (DRF-2018-11-ST2-048). ACT is supported by a Tier 2 Canada Research Chair in Knowledge Synthesis. DM is supported in part by a University Research Chair, University of Ottawa. The funders had no role in considering the study design or in the collection, analysis, interpretation of data, writing of the report, or decision to submit the article for publication.

Competing interests: All authors have completed the ICMJE uniform disclosure form at http://www.icmje.org/conflicts-of-interest/ and declare: EL is head of research for the *BMJ*; MJP is an editorial board member for *PLOS Medicine*; ACT is an associate editor and MJP, TL, EMW, and DM are editorial board members for the *Journal of Clinical Epidemiology*; DM and LAS were editors in chief, LS, JMT, and ACT are associate editors, and JG is an editorial board member for *Systematic Reviews*. None of these authors were involved in the peer review process or decision to publish. TCH has received personal fees from Elsevier outside the submitted work. EMW has received personal fees from the *American Journal for Public Health*, for which he is the editor for systematic reviews. VW is editor in chief of the Campbell Collaboration, which produces systematic reviews, and co-convenor of the Campbell and Cochrane equity methods group. DM is chair of the EQUATOR Network, IB is adjunct director of the French EQUATOR Centre and TCH is co-director of the Australasian EQUATOR Centre, which advocates for the use of reporting guidelines to improve the quality of reporting in research articles. JMT received salary from Evidence Partners, creator of DistillerSR software for systematic reviews; Evidence Partners was not involved in the design or outcomes of the statement, and the views expressed solely represent those of the author.

Provenance and peer review: Not commissioned; externally peer reviewed.

BMJ: first published as 10.1136/bmj.n71 on 29 March 2021. Downloaded from https://www.bmj.com/ on 16 February 2026 by guest. Protected by copyright, including for uses related to text and data mining, AI training, and similar technologies.

**Patient and public involvement:** Patients and the public were not involved in this methodological research. We plan to disseminate the research widely, including to community participants in evidence synthesis organisations.

This is an Open Access article distributed in accordance with the terms of the Creative Commons Attribution (CC BY 4.0) license, which permits others to distribute, remix, adapt and build upon this work, for commercial use, provided the original work is properly cited. See: http://creativecommons.org/licenses/by/4.0/.

1 Gurevitch J, Koricheva J, Nakagawa S, Stewart G. Meta-analysis and the science of research synthesis. *Nature* 2018;555:175-82. doi:10.1038/nature25753
2 Gough D, Thomas J, Oliver S. Clarifying differences between reviews within evidence ecosystems. *Syst Rev* 2019;8:170. doi:10.1186/s13643-019-1089-2
3 Moher D. Reporting guidelines: doing better for readers. *BMC Med* 2018;16:233. doi:10.1186/s12916-018-1226-0
4 Moher D, Liberati A, Tetzlaff J, Altman DG, PRISMA Group. Preferred reporting items for systematic reviews and meta-analyses: the PRISMA statement. *Ann Intern Med* 2009;151:264-9, W64. doi:10.7326/0003-4819-151-4-200908180-00135
5 Moher D, Liberati A, Tetzlaff J, Altman DG, PRISMA Group. Preferred reporting items for systematic reviews and meta-analyses: the PRISMA statement. *BMJ* 2009;339:b2535. doi:10.1136/bmj.b2535
6 Moher D, Liberati A, Tetzlaff J, Altman DG, PRISMA Group. Preferred reporting items for systematic reviews and meta-analyses: the PRISMA statement. *PLoS Med* 2009;6:e1000097. doi:10.1371/journal.pmed.1000097
7 Moher D, Liberati A, Tetzlaff J, Altman DG, PRISMA Group. Preferred reporting items for systematic reviews and meta-analyses: the PRISMA statement. *J Clin Epidemiol* 2009;62:1006-12. doi:10.1016/j.jclinepi.2009.06.005
8 Moher D, Liberati A, Tetzlaff J, Altman DG, PRISMA Group. Preferred reporting items for systematic reviews and meta-analyses: the PRISMA statement. *Int J Surg* 2010;8:336-41. doi:10.1016/j.ijsu.2010.02.007
9 Moher D, Liberati A, Tetzlaff J, Altman DG, PRISMA Group. Preferred reporting items for systematic reviews and meta-analyses: the PRISMA Statement. *Open Med* 2009;3:e123-30.
10 Moher D, Liberati A, Tetzlaff J, Altman DG, PRISMA Group. Reprint--preferred reporting items for systematic reviews and meta-analyses: the PRISMA statement. *Phys Ther* 2009;89:873-80. doi:10.1093/ptj/89.9.873
11 Moher D, Tetzlaff J, Tricco AC, Sampson M, Altman DG. Epidemiology and reporting characteristics of systematic reviews. *PLoS Med* 2007;4:e78. doi:10.1371/journal.pmed.0040078
12 Liberati A, Altman DG, Tetzlaff J, et al. The PRISMA statement for reporting systematic reviews and meta-analyses of studies that evaluate health care interventions: explanation and elaboration. *J Clin Epidemiol* 2009;62:e1-34. doi:10.1016/j.jclinepi.2009.06.006
13 Liberati A, Altman DG, Tetzlaff J, et al. The PRISMA statement for reporting systematic reviews and meta-analyses of studies that evaluate healthcare interventions: explanation and elaboration. *BMJ* 2009;339:b2700. doi:10.1136/bmj.b2700
14 Liberati A, Altman DG, Tetzlaff J, et al. The PRISMA statement for reporting systematic reviews and meta-analyses of studies that evaluate health care interventions: explanation and elaboration. *Ann Intern Med* 2009;151:W65-94. doi:10.7326/0003-4819-151-4-200908180-00136
15 Liberati A, Altman DG, Tetzlaff J, et al. The PRISMA statement for reporting systematic reviews and meta-analyses of studies that evaluate health care interventions: explanation and elaboration. *PLoS Med* 2009;6:e1000100. doi:10.1371/journal.pmed.1000100
16 Liberati A, Altman DG, Tetzlaff J, et al. The PRISMA statement for reporting systematic reviews and meta-analyses of studies that evaluate health care interventions: explanation and elaboration. *PLoS Med* 2009;6:e1000100. doi:10.1371/journal.pmed.1000100
17 Page MJ, Shamseer L, Altman DG, et al. Epidemiology and reporting characteristics of systematic reviews of biomedical research: a cross-sectional study. *PLoS Med* 2016;13:e1002028. doi:10.1371/journal.pmed.1002028
18 Panic N, Leoncini E, de Belvis G, Ricciardi W, Boccia S. Evaluation of the endorsement of the preferred reporting items for systematic reviews and meta-analysis (PRISMA) statement on the quality of published systematic review and meta-analyses. *PLoS One* 2013;8:e83138. doi:10.1371/journal.pone.0083138
19 Agha RA, Fowler AJ, Limb C, et al. Impact of the mandatory implementation of reporting guidelines on reporting quality in a surgical journal: A before and after study. *Int J Surg* 2016;30:169-72. doi:10.1016/j.ijsu.2016.04.032
20 Leclercq V, Beaudart C, Ajamieh S, Rabenda V, Tirelli E, Bruyère O. Meta-analyses indexed in PsycINFO had a better completeness of reporting when they mention PRISMA. *J Clin Epidemiol* 2019;115:46-54. doi:10.1016/j.jclinepi.2019.06.014
21 Page MJ, Moher D. Evaluations of the uptake and impact of the Preferred Reporting Items for Systematic reviews and Meta-Analyses (PRISMA) Statement and extensions: a scoping review. *Syst Rev* 2017;6:263. doi:10.1186/s13643-017-0663-8
22 O'Mara-Eves A, Thomas J, McNaught J, Miwa M, Ananiadou S. Using text mining for study identification in systematic reviews: a systematic review of current approaches. *Syst Rev* 2015;4:5. doi:10.1186/2046-4053-4-5
23 Marshall IJ, Noel-Storr A, Kuiper J, Thomas J, Wallace BC. Machine learning for identifying randomized controlled trials: an evaluation and practitioner's guide. *Res Synth Methods* 2018;9:602-14. doi:10.1002/jrsm.1287
24 Marshall IJ, Wallace BC. Toward systematic review automation: a practical guide to using machine learning tools in research synthesis. *Syst Rev* 2019;8:163. doi:10.1186/s13643-019-1074-9
25 McKenzie JE, Brennan SE. Synthesizing and presenting findings using other methods. In: Higgins JPT, Thomas J, Chandler J, et al, eds. *Cochrane Handbook for Systematic Reviews of Interventions* . Cochrane, 2019. doi:10.1002/9781119536604.ch12 .
26 Higgins JPT, López-López JA, Becker BJ, et al. Synthesising quantitative evidence in systematic reviews of complex health interventions. *BMJ Glob Health* 2019;4(Suppl 1):e000858. doi:10.1136/bmjgh-2018-000858
27 Campbell M, McKenzie JE, Sowden A, et al. Synthesis without meta-analysis (SWiM) in systematic reviews: reporting guideline. *BMJ* 2020;368:l6890. doi:10.1136/bmj.l6890
28 Sterne JAC, Savović J, Page MJ, et al. RoB 2: a revised tool for assessing risk of bias in randomised trials. *BMJ* 2019;366:l4898. doi:10.1136/bmj.l4898
29 Sterne JA, Hernán MA, Reeves BC, et al. ROBINS-I: a tool for assessing risk of bias in non-randomised studies of interventions. *BMJ* 2016;355:i4919. doi:10.1136/bmj.i4919
30 Whiting P, Savović J, Higgins JP, et al, ROBIS group. ROBIS: A new tool to assess risk of bias in systematic reviews was developed. *J Clin Epidemiol* 2016;69:225-34. doi:10.1016/j.jclinepi.2015.06.005
31 Shea BJ, Reeves BC, Wells G, et al. AMSTAR 2: a critical appraisal tool for systematic reviews that include randomised or non-randomised studies of healthcare interventions, or both. *BMJ* 2017;358:j4008. doi:10.1136/bmj.j4008
32 Hultcrantz M, Rind D, Akl EA, et al. The GRADE Working Group clarifies the construct of certainty of evidence. *J Clin Epidemiol* 2017;87:4-13. doi:10.1016/j.jclinepi.2017.05.006
33 Booth A, Clarke M, Dooley G, et al. The nuts and bolts of PROSPERO: an international prospective register of systematic reviews. *Syst Rev* 2012;1:2. doi:10.1186/2046-4053-1-2
34 Moher D, Stewart L, Shekelle P. Establishing a new journal for systematic review products. *Syst Rev* 2012;1:1. doi:10.1186/2046-4053-1-1
35 Page MJ, McKenzie JE, Bossuyt PM, et al. Updating guidance for reporting systematic reviews: development of the PRISMA 2020 statement. *J Clin Epidemiol* 2021;S0895-4356(21)00040-8. doi:10.1016/j.jclinepi.2021.02.003.
36 Page MJ, Altman DG, Shamseer L, et al. Reproducible research practices are underused in systematic reviews of biomedical interventions. *J Clin Epidemiol* 2018;94:8-18. doi:10.1016/j.jclinepi.2017.10.017
37 Page MJ, Altman DG, McKenzie JE, et al. Flaws in the application and interpretation of statistical analyses in systematic reviews of therapeutic interventions were common: a cross-sectional analysis. *J Clin Epidemiol* 2018;95:7-18. doi:10.1016/j.jclinepi.2017.11.022
38 Page MJ, McKenzie JE, Bossuyt PM, et al. Mapping of reporting guidance for systematic reviews and meta-analyses generated a comprehensive item bank for future reporting guidelines. *J Clin Epidemiol* 2020;118:60-8. doi:10.1016/j.jclinepi.2019.11.010
39 Tong A, Flemming K, McInnes E, Oliver S, Craig J. Enhancing transparency in reporting the synthesis of qualitative research: ENTREQ. *BMC Med Res Methodol* 2012;12:181. doi:10.1186/1471-2288-12-181
40 France EF, Cunningham M, Ring N, et al. Improving reporting of meta-ethnography: the eMERGe reporting guidance. *BMC Med Res Methodol* 2019;19:25. doi:10.1186/s12874-018-0600-0
41 Page MJ, Moher D, Bossuyt PM, et al. PRISMA 2020 explanation and elaboration: updated guidance and exemplars for reporting systematic reviews.*BMJ* 2021;372:n160. doi:10.1136/bmj.n160.
42 Rethlefsen ML, Kirtley S, Waffenschmidt S, et al, PRISMA-S Group. PRISMA-S: an extension to the PRISMA statement for reporting literature searches in systematic reviews. *Syst Rev* 2021;10:39. doi:10.1186/s13643-020-01542-z.
43 Higgins JPT, Thomas J, Chandler J eds. *Cochrane Handbook for Systematic Reviews of Interventions*: Version 6.0. Cochrane, 2019. Available from https://training.cochrane.org/handbook.
44 Dekkers OM, Vandenbroucke JP, Cevallos M, Renehan AG, Altman DG, Egger M. COSMOS-E: Guidance on conducting systematic reviews and meta-analyses of observational studies of etiology. *PLoS Med* 2019;16:e1002742. doi:10.1371/journal.pmed.1002742

BMJ: first published as 10.1136/bmj.n71 on 29 March 2021. Downloaded from https://www.bmj.com/ on 16 February 2026 by guest. Protected by copyright, including for uses related to text and data mining, AI training, and similar technologies.

45 Cooper H, Hedges LV, Valentine JV, eds. *The Handbook of Research Synthesis and Meta-Analysis*. Russell Sage Foundation, 2019.
46 IOM (Institute of Medicine). *Finding What Works in Health Care: Standards for Systematic Reviews*. The National Academies Press, 2011.
47 Moher D, Shamseer L, Clarke M, et al, PRISMA-P Group. Preferred reporting items for systematic review and meta-analysis protocols (PRISMA-P) 2015 statement. *Syst Rev* 2015;4:1. doi:10.1186/2046-4053-4-1
48 Shamseer L, Moher D, Clarke M, et al, PRISMA-P Group. Preferred reporting items for systematic review and meta-analysis protocols (PRISMA-P) 2015: elaboration and explanation. *BMJ* 2015;350:g7647. doi:10.1136/bmj.g7647
49 Hutton B, Salanti G, Caldwell DM, et al. The PRISMA extension statement for reporting of systematic reviews incorporating network meta-analyses of health care interventions: checklist and explanations. *Ann Intern Med* 2015;162:777-84. doi:10.7326/M14-2385
50 Stewart LA, Clarke M, Rovers M, et al, PRISMA-IPD Development Group. Preferred Reporting Items for Systematic Review and Meta-Analyses of individual participant data: the PRISMA-IPD Statement. *JAMA* 2015;313:1657-65. doi:10.1001/jama.2015.3656
51 Zorzela L, Loke YK, Ioannidis JP, et al, PRISMAHarms Group. PRISMA harms checklist: improving harms reporting in systematic reviews. *BMJ* 2016;352:i157. doi:10.1136/bmj.i157
52 McInnes MDF, Moher D, Thombs BD, et al, and the PRISMA-DTA Group. Preferred reporting items for a systematic review and meta-analysis of diagnostic test accuracy studies: the PRISMA-DTA statement. *JAMA* 2018;319:388-96. doi:10.1001/jama.2017.19163
53 Tricco AC, Lillie E, Zarin W, et al. PRISMA extension for scoping reviews (PRISMA-SCR): Checklist and explanation. *Ann Intern Med* 2018;169:467-73. doi:10.7326/M18-0850
54 Beller EM, Glasziou PP, Altman DG, et al, PRISMA for Abstracts Group. PRISMA for Abstracts: reporting systematic reviews in journal and conference abstracts. *PLoS Med* 2013;10:e1001419. doi:10.1371/journal.pmed.1001419
55 Boers M. Graphics and statistics for cardiology: designing effective tables for presentation and publication. *Heart* 2018;104:192-200. doi:10.1136/heartjnl-2017-311581
56 Mayo-Wilson E, Li T, Fusco N, Dickersin KMUDS investigators. Practical guidance for using multiple data sources in systematic reviews and meta-analyses (with examples from the MUDS study). *Res Synth Methods* 2018;9:2-12. doi:10.1002/jrsm.1277
57 Stovold E, Beecher D, Foxlee R, Noel-Storr A. Study flow diagrams in Cochrane systematic review updates: an adapted PRISMA flow diagram. *Syst Rev* 2014;3:54. doi:10.1186/2046-4053-3-54
58 McGuinness LA. mcguinlu/PRISMA-Checklist: Initial release for manuscript submission (Version v1.0.0).*Zenodo* . doi:10.5281/zenodo.3994319. 2020.
59 Aczel B, Szaszi B, Sarafoglou A, et al. A consensus-based transparency checklist. *Nat Hum Behav* 2020;4:4-6. doi:10.1038/s41562-019-0772-6
60 Barnes C, Boutron I, Giraudeau B, Porcher R, Altman DG, Ravaud P. Impact of an online writing aid tool for writing a randomized trial report: the COBWEB (Consort-based WEB tool) randomized controlled trial. *BMC Med* 2015;13:221. doi:10.1186/s12916-015-0460-y
61 Chauvin A, Ravaud P, Moher D, et al. Accuracy in detecting inadequate research reporting by early career peer reviewers using an online CONSORT-based peer-review tool (COBPeer) versus the usual peer-review process: a cross-sectional diagnostic study. *BMC Med* 2019;17:205. doi:10.1186/s12916-019-1436-0
62 Wayant C, Page MJ, Vassar M. Evaluation of reproducible research practices in oncology systematic reviews with meta-analyses referenced by national comprehensive cancer network guidelines. *JAMA Oncol* 2019;5:1550-5. doi:10.1001/jamaoncol.2019.2564
63 McKenzie JE, Brennan SE. Overviews of systematic reviews: great promise, greater challenge. *Syst Rev* 2017;6:185. doi:10.1186/s13643-017-0582-8
64 Moher D, Schulz KF, Simera I, Altman DG. Guidance for developers of health research reporting guidelines. *PLoS Med* 2010;7:e1000217. doi:10.1371/journal.pmed.1000217
65 Simera I, Moher D, Hirst A, Hoey J, Schulz KF, Altman DG. Transparent and accurate reporting increases reliability, utility, and impact of your research: reporting guidelines and the EQUATOR Network. *BMC Med* 2010;8:24. doi:10.1186/1741-7015-8-24
66 Speich B, Schroter S, Briel M, et al. Impact of a short version of the CONSORT checklist for peer reviewers to improve the reporting of randomised controlled trials published in biomedical journals: study protocol for a randomised controlled trial. *BMJ Open* 2020;10:e035114. doi:10.1136/bmjopen-2019-035114
67 Stevens A, Shamseer L, Weinstein E, et al. Relation of completeness of reporting of health research to journals' endorsement of reporting guidelines: systematic review. *BMJ* 2014;348:g3804. doi:10.1136/bmj.g3804
68 Hair K, Macleod MR, Sena ESIICARus Collaboration. A randomised controlled trial of an Intervention to Improve Compliance with the ARRIVE guidelines (IICARus). *Res Integr Peer Rev* 2019;4:12. doi:10.1186/s41073-019-0069-3
69 Blanco D, Altman D, Moher D, Boutron I, Kirkham JJ, Cobo E. Scoping review on interventions to improve adherence to reporting guidelines in health research. *BMJ Open* 2019;9:e026589. doi:10.1136/bmjopen-2018-026589
70 Charters E. The use of think-aloud methods in qualitative research: an introduction to think-aloud methods. *Brock Education Journal* 2003;12. doi:10.26522/brocked.v12i2.38 .
71 Welch V, Petticrew M, Tugwell P, et al, PRISMA-Equity Bellagio group. PRISMA-Equity 2012 extension: reporting guidelines for systematic reviews with a focus on health equity. *PLoS Med* 2012;9:e1001333. doi:10.1371/journal.pmed.1001333
72 Wang X, Chen Y, Liu Y, et al. Reporting items for systematic reviews and meta-analyses of acupuncture: the PRISMA for acupuncture checklist. *BMC Complement Altern Med* 2019;19:208. doi:10.1186/s12906-019-2624-3


PRISMA 2020 checklist
PRISMA 2020 expanded checklist

BMJ: first published as 10.1136/bmj.n71 on 29 March 2021. Downloaded from https://www.bmj.com/ on 16 February 2026 by guest. Protected by copyright, including for uses related to text and data mining, AI training, and similar technologies.
